# Laboratory Characterization of Porous Asphalt Mixtures with Aramid Fibers

**DOI:** 10.3390/ma14081935

**Published:** 2021-04-13

**Authors:** Anik Gupta, Pedro Lastra-Gonzalez, Daniel Castro-Fresno, Jorge Rodriguez-Hernandez

**Affiliations:** GITECO Research Group, University of Cantabria, 39005 Santander, Spain; pedro.lastragonzalez@unican.es (P.L.-G.); castrod@unican.es (D.C.-F.); jorge.rodriguez@unican.es (J.R.-H.)

**Keywords:** aramid fibers, porous asphalt mixtures, fracture energy, toughness, open-graded mixtures

## Abstract

Recent studies have shown that fibers improve the performance of porous asphalt mixtures. In this study, the influence of four different fibers, (a) regular aramid fiber (RegAR), (b) aramid fiber with latex coating (ARLat), (c) aramid fiber with polyurethane coating (ARPoly), (d) aramid fiber of length 12 mm (AR12) was evaluated on abrasion resistance and toughness of the mixtures. The functional performance was estimated using permeability tests and the mechanical performance was evaluated using the Cantabro test and indirect tensile strength tests. The parameters such as fracture energy, post cracking energy, and toughness were obtained through stress-strain plots. Based on the analysis of results, it was concluded that the addition of ARLat fibers enhanced the abrasion resistance of the mixtures. In terms of ITS, ARPoly and RegAR have positively influenced mixtures under dry conditions. However, the mixtures with all aramid fibers were found to have adverse effects on the ITS under wet conditions and energy parameters of porous asphalt mixtures with the traditional percentages of bitumen in the mixture used in Spain (i.e., approximately 4.5%).

## 1. Introduction

### 1.1. Background

The aggregate gradation design in porous asphalt mixtures requires a lower amount of fine aggregates, which results in high permeability and lower integrity of the structure compared to conventional dense-graded mixtures [1,2]. When combined with environmental factors and traffic load, there is an increment in the oxidation of bitumen in the open-graded structure, which results in stiffening of the mixtures [3]. Further, the aggregates start loosening contact that leads to raveling. It is speculated that at low and high temperatures, raveling occurs due to fracture and lack of strength while at intermediate temperatures, this occurs due to fatigue failure [1]. Owing to their poor durability, the advantages of porous asphalt mixtures such as better skid resistance, noise reduction, etc. are overshadowed [4,5,6]. Consequently, researchers continuously aim to improve the durability and strength of porous asphalt (PA) mixtures. 

The incorporation of modified binders and various additives into PA mixtures may improve the mastic and the integrity of the structure [7,8]. Malarvizhi et al. [9] used crumb-rubber-modified bitumen and polymer-modified bitumen to improve the raveling resistance of porous asphalt mixtures. Landi et al. [10] utilized the life-cycle assessment to analyze the environmental impact of three PA mixtures: control, cellulose-reinforced, and end-of-life (ELT) fiber-reinforced. It was found that ELT fibers had the least impact on the environment. Additionally, they had shown remarkable improvement in the fatigue performance of PA mixtures as compared to cellulose fibers. Masri et al. [11] utilized nano-silica to resist the damage caused by moisture. Warm mix additives are also known to improve the performance of PA mixtures. In a study by Cheng et al. [12], it was concluded that warm-mix PA mixtures have better marshal stability and permeability than hot-mix PA. Shukry et al. [13] tested various fillers such as hydrated lime, cement, and diatomite in open-graded mixtures and performed field emission scanning electron microscope (FESEM) and energy dispersive X-ray (EDX) analyses. It was concluded that the permeability rates are not compromised by incorporating these fillers. On one hand, Diatomite filler enhances the rutting resistance, moisture damage, resilient modulus, and indirect tensile strength, on the other, hydrated lime improved the cohesion and reduced particle loss. Haryati et al. [14] added coconut shells to PA mixtures as substitutes to aggregates and fibers as additions. It was concluded that coconut shells and fibers improve stability and rutting resistance. While in another study [15], coconut shells and plastic bottle waste were analyzed as fillers in PA mixtures. It was suggested that the plastic bottle waste improved the binder drainage and coconut shells, in agreement with the previous study [16] enhanced the Marshal stability of PA mixtures. Skaf et al., 2019 [17] investigated the benefits of adding electric arc furnace slag and found that their use improved the permeability and tensile strength of PA mixtures, but their addition could reduce the abrasion resistance. In another study [18], hybrid glass fibers were added in combination with nano-silica, the authors suggest that the addition of 0.2% glass fiber and 0.3% of polypropylene fiber content resulted in minimum rutting.

### 1.2. Aramid Fibers

Aramid fibers are synthetic fibers with long-chain synthetic polyamide and more than 85% of amid are linked directly to aromatic rings [18,19]. These types of fiber offer high resistance towards heat and organic solvents and they degrade at a very high temperature of 500 °C [20]. Due to their high melting point, the aramid fibers do not undergo any degradation at the manufacturing temperature of asphalt mixtures [21]. Many studies have been conducted on the use of aramid fibers to enhance the mechanical, economical, and environmental aspects of asphalt mixtures owing to their remarkable properties [22,23,24,25,26,27,28,29,30]. Xing et al. [31] investigated the effect of composites in asphalt binders by assessing rheological parameters and micromorphology using a scanning electron microscope. It was found that aramid fibers reinforced the asphalt mixture and enhanced the strain recovery rate in multiple stress creep recovery tests (MSCRT). Badeli [32] added aramid pulp fibers to the dense-graded asphalt mixtures and found that these fibers improve the stiffness at medium and high temperatures. Furthermore, aramid fibers reinforce the asphalt mixtures so their addition results in improvement of fracture toughness and fracture energy [33,34]. Xing et al. [35] mentioned that the binder adsorption of the fiber reflects the stabilizing ability of the fiber. It was pointed out that aramid fibers absorb more binder due to their complex bundle shape as compared to basalt fibers, however, their binder absorption was lower than the lignin fibers. Kassem et al. [25] studied the effect of fibers using the viscoelastoplastic continuum damage (VEPCD) model. The authors stated that warm mix additive-enhanced the dispersion of aramid fibers in asphalt concrete and improved its rutting resistance. In another study [36], the author suggested that aramid fiber improves the stiffness and fatigue life of asphalt mixtures at low-frequency loads (high-speed traffic conditions). Limited literature was available on the use of aramid fibers in porous asphalt mixtures (<10 articles in the last decade). Gupta et al. [37] incorporated aramid and glass fibers into porous asphalt mixtures and concluded that the aramid fibers showed remarkable improvement in abrasion resistance. Slebi-Acevedo et al. [38], conducted research on polyacrinite and polyolefin plus aramid fibers in porous asphalt mixtures. It was found that the use of polyolefin plus aramid fibers enhanced abrasion resistance, reduced binder drainage, and improved the toughness of porous asphalt mixtures. In another study [39], the influence of fiber was found to be more prominent in the presence of conventional bitumen as compared to polymer-modified bitumen. Aramid fibers are not commonly used in porous asphalt mixtures, but they have an important potential to improve PA as their use can act as reinforcement in the binder-aggregate matrix, which may result in high resistance towards abrasion loss and fatigue cracking.

### 1.3. Objective and Methodology

Considering the limited literature available on the use of aramid fibers in PA mixtures, the primary objective of this study is to evaluate the effect of four different fibers on the functional and mechanical characteristics of porous asphalt mixtures. The laboratory tests include permeability test, air voids analysis, Cantabro test, indirect tensile strength (ITS) test. Additionally, energy parameters such as fracture energy (FE), post-cracking energy (PE), and toughness were analyzed to obtain wider information on mixture properties. The bitumen content was designed on the criteria of minimum binder drainage and maximum abrasion resistance, but without considering the anti-drainage capacity of fibers, so their capacity to strengthen the mixtures was tested with the same quantity of binder as of the reference mixture. It is worth mentioning here that the aim is to study the effect of fibers without altering the binder quantity, to compare the influence of fibers on the mechanical resistance.

## 2. Materials and Methods

**Figure 1:**
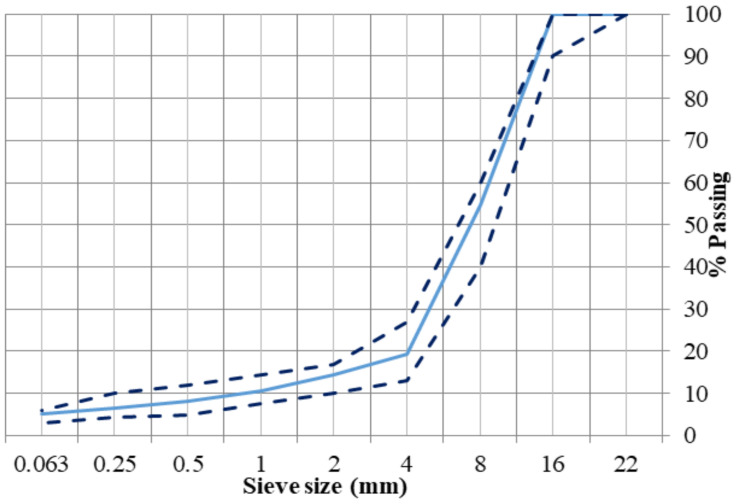
Gradation of PA mixtures.

**Figure 2:**
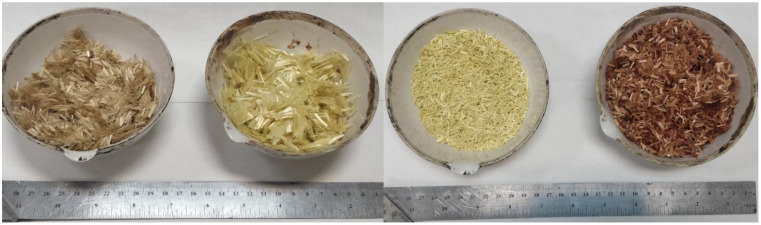
Fibers used in the study from left to right: RegAR, AR12, ARPoly, and ARLat.

Porous asphalt mixtures were prepared according to the Spanish guidelines [40] with 16 mm as the maximum size of aggregates. The size distribution of all the aggregates is according to Figure 1. The minimum requirement of the air void content for porous asphalt mixtures is 20%. The properties of the bitumen are given in Table 1 and that of fibers and aggregates in Table 2, respectively. The volumetric analysis was done according to the European standard EN 12697-8. The bitumen content of 4.5% and fiber content of 0.05% was fixed based on the previous study by the authors [36]. Virgin binder of penetration grade 50/70 and commercially used Polymer-Modified Bitumen (PMB 45/80-65) were used to prepare the mixtures. Table 3 represents the mixture types prepared in the study. Figure 2 shows the four commercial fibers used, which were regular aramid fibers of two different sizes (6 mm and 12 mm) and aramid fibers with two coatings (latex and polyurethane). The idea was to compare the mixtures under the same conditions and check whether fibers improve the properties of mixtures by themselves, without increasing the percentage of bitumen. Therefore, high binder content was not used. Due to a high volume of air voids, the binder drainage and abrasion loss are significant factors that determine the performance of the porous asphalt mixtures. 

Therefore, the mixtures in this study were designed based on the requirement of air void content of 20%, with minimum binder drainage and minimum abrasion loss criteria. It was observed that for bitumen content of 4.5% and fiber content of 0.05%, binder drainage was not a problem. For all the mixtures, draindown was around 0.01% according to European standard EN 12697-18, which is clearly under the permissible limits of 0.3% [2,41,42,43]. Reference mixtures were designed with virgin bitumen with a binder content of 4.5% with no fiber. One more mixture with polymer-modified bitumen was also prepared to check the level of improvement of the experimental mixtures. The binder content and fiber content are kept constant to analyze the effect of fiber exclusively. In this paper, the aim is to compute the effect of different types of aramid fibers on the mechanical strength of the porous asphalt mixtures; therefore, it was ensured that the abrasion resistance did not surpass the permissible limits. 

### 2.1. Permeability Test

The permeability of porous asphalt mixtures is an important property to ensure appropriate drainage of the mixture. Therefore, while any additive added to the mixture should not reduce the permeability of the mixture considerably. The permeability of the samples was measured with a flow falling head permeameter as performed by various researchers [44,45,46]. The permeability is calculated based on Darcy’s law (Equation (1)).

(1) $$k\;=\;2.3\frac{\mathrm{aL}}{\mathrm{At}}\left[\log\left(\frac{h_{1}}{h_{2}}\right)\right]$$

where a and A are the cross-sections of the standpipe and the specimen (mm^2^) respectively; t is the time required for the water to fall from an initial height of 300 mm above the sample (h_1_) to a height of 100 mm above the sample (h_2_), and L is the height of the specimen (mm). As a reference for comparison, the minimum values of permeability reported by many researchers are given in Table 4.

### 2.2. Cantabro Test

The Cantabro test is conducted to measure the abrasion resistance of porous asphalt mixtures. In this study, this test was done under dry conditions according to EN 12697–17 and under wet conditions according to Spanish guidelines NLT 362/92. In wet conditions, the samples are kept in water at 60 °C temperature for 24 h. Then the samples are kept at 25 °C for 24 h before the test. The particle loss should not exceed 20% in the case of dry conditions and 35% under wet conditions for the most restrictive level. The particle loss is calculated using Equation (2).

(2) $$\mathrm{Particle}\;\mathrm{loss}\;(\%)=\left(\frac{\mathrm{Initial}\;\mathrm{mass}-\;\mathrm{Final}\;\mathrm{mass}}{\mathrm{Initial}\;\mathrm{mass}}\right)\times 100$$

### 2.3. Indirect Tensile Strength Test

The indirect tensile strength is computed according to EN 12697-23 under dry and wet conditions. Four replicates were prepared for each dry and wet condition using a Marshal Compactor. The specimens under dry conditions were kept at ambient temperature for 24 h followed by 3 h at the test temperature in a conditioning chamber. A material testing system with a maximum capacity of 100 kN is utilized to determine the ITS. The test was done at a constant rate displacement of 50 mm/min. For the wet conditions, the samples were kept in distilled water for 30 min in a vacuum vessel filled at ambient temperature then were kept for 68–72 h at 40 °C. The replicates of all mixture types were then placed at 15 °C for 3 h before breaking every sample. The maximum load at which the failure occurs is the indirect strength of the sample. Indirect Tensile Strength Ratio (ITSR) is representative of the moisture susceptibility of the mixture, low ITSR signifies that the samples are more sensitive to the presence of moisture. The Indirect Tensile Strength Ratio (ITSR) is calculated according to Equation (3), based on EN 12697-12. A minimum ITSR of 85% is required according to Spanish guidelines [39].

(3) $$\mathrm{ITSR}=100\times \frac{{\mathrm{ITS}}_{w}}{{\mathrm{ITS}}_{d}}$$

where ITS_w_ is the Indirect Tensile Strength of moisture-conditioned sample specimens and ITS_d_ is the Indirect Tensile Strength of unconditioned specimens.

Based on stress-strain plots obtained from the Indirect tensile test, the fracture toughness of the mixtures was calculated. Fracture toughness represents the resistance to fracture of any material when there are initial cracks in the body [52]. The toughness of the samples is the area under the stress vs. strain curve, which is the sum of Fracture Energy (F.E) and Post-cracking Energy (P.E). Fracture energy is the area under the curve until the maximum stress (σ_max_) is achieved, and it represents the cracking resistance of the sample. The post cracking energy represents the ductility of the pavement, and it is the area under the curve from the vertical strain at which maximum stress is achieved until double that strain (from ε_max_ to 2ε_max_). To calculate the toughness, for every load level, the displacement incurred is recorded by a data acquisition system. The stresses are calculated with Equation (4).

(4) $$\mathrm{Stress}\;(\sigma )=\frac{2P}{\pi \mathrm{Dt}}$$

where P is the load (kN), D is the diameter of the specimen (mm), t is the thickness of the specimen (mm) and D is the initial diameter of the specimen.

### 2.4. Statistical Analysis

The accuracy of the results was confirmed by statistical analysis. The Anderson–Darling normality test was used to check the normality and homoscedasticity of the data. Data that follow normal distribution are analyzed by one-way analysis of variance (ANOVA). For the non-normal data, non-parametric Kruskal–Wallis tests were performed. The statistical analysis tests were performed with a confidence level of 95%. That means that if the *p*-value is less than 0.05, the null hypothesis is rejected.

## 3. Results and Discussion

### 3.1. Air Voids and Permeability

**Figure 3:**
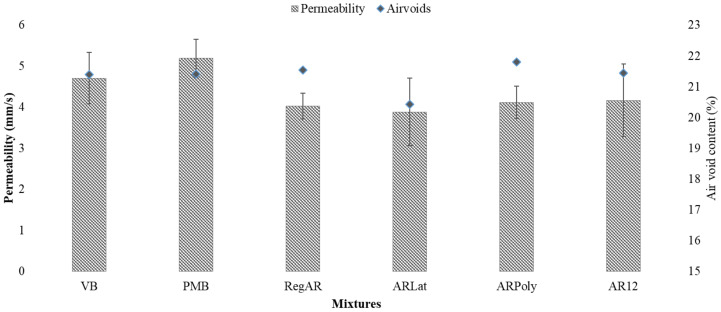
Permeability and air voids of the mixtures, error bars indicate the standard deviation about the mean.

The results for air voids and permeability are given in Figure 3. The air void content of all the mixtures is found to be higher than 20%. ARLat has the highest air void content. However, no significant reduction was observed when the ANOVA results were analyzed: the mixtures shared the same group as the *p*-value was greater than 0.05. Consequently, the percentage of voids can be considered practically the same. This can be explained by the fact that the fiber content used in the study was low (0.05%). 

The permeability of the PA samples displays the ability to transmit water. The addition of fibers has a negligible influence on the permeability of PA mixtures as well. However, the plot indicates slight reductions in permeability, which can be because fibers block part of the air voids in the mixtures. However, the error bar suggests that the range of permeability of all mixture types is the same. Moreover, all mixtures pass the minimum requirements of permeability as suggested by various researchers in Table 4. 

### 3.2. Cantabro Test

**Figure 4:**
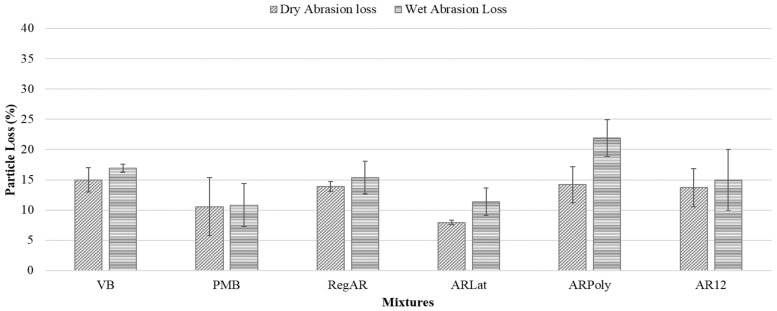
Particle loss by Cantabro test, error bars indicate the standard deviation about the mean.

Cantabro test determines the raveling potential of porous asphalt mixtures, higher particle loss signifies a higher degree of expected raveling in the pavement. The particle loss of the mixtures under dry and wet conditions is shown in Figure 4. It can be observed that the fibers show a positive influence on the abrasion resistance by reducing the particle loss as compared to the control mixture VB under dry conditions. The highest improvement is observed in the case of ARLat fibers, these fibers have significantly reduced the particle loss of porous asphalt mixtures (46.7%). ARLat displays even better abrasion resistance than PMB mixtures, which may be due to the latex coating that resulted in improved cohesion in the asphalt matrix. However, other aramid fibers were not able to match the performance of the PMB mixtures. 

Under wet conditions, all aramid fibers except ARPoly showed better results than the reference mixture. The percentage improvement and reduction in abrasion resistance for each fiber as compared to VB and PMB can be found in Table 5. ARLat fibers improve abrasion resistance considerably as compared to the VB mixture (32.5%). However, the only fiber that significantly reduced the abrasion resistance under wet conditions is ARPoly fiber, this can be attributed to the high moisture sensitivity of the ARPoly fiber or due to lower binder content. 

### 3.3. Indirect Tensile Strength Test

**Figure 5:**
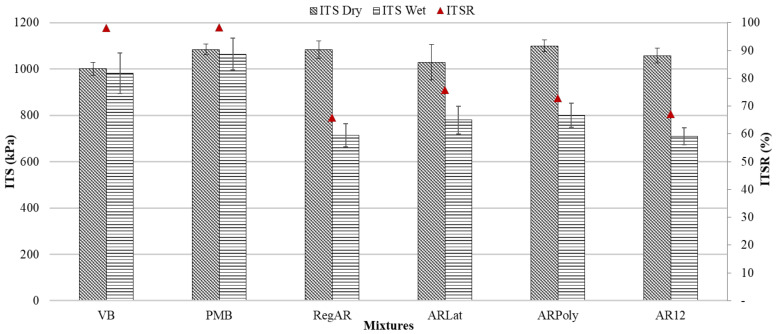
Average values of indirect tensile strength, error bars indicate the standard deviation about the mean.

The results of the indirect tensile strength test under dry and wet conditions are given in Figure 5. Under dry conditions, fibers increase the ITS of the mixtures. Regular aramid fibers (RegAR) increase the ITS by 8.33% to the reference with the virgin binder (VB), while their strength is similar to PMB. The ARPoly fibers also improved the ITS (10%) as compared to VB while aramid fiber with 12 mm length (AR12) and with latex coating (ARLat) do not display significant variation from the reference mixture. It can be concluded that all aramid fibers have a positive influence on the ITS of porous asphalt mixtures under dry conditions, some of them even reach the same level as polymer-modified bitumen. 

The percentage variation with respect to the reference mixture and ANOVA analysis for the indirect tensile strength of mixtures under dry and wet conditions are given in Table 6. Under wet conditions, the variation in the ITS is significant, the *p*-value is less than 0.05, which implies that the null hypothesis is rejected, and the difference is statistically significant. Consequently, the strength of mixtures with fibers under wet conditions is less than the reference mixture and PMB mixture. 

This may be attributed to the high susceptibility of the fibers to moisture. The highest reduction was seen in the case of AR12, followed by RegAR with a similar reduction as both these fibers are without coating. The fibers with coating display better performance compared to regular aramid fibers of 6 mm and 12 mm length, although the difference is small. Therefore, the presence of coating may resist the penetration of moisture. 

For all aramid fibers, the ITSR fails the permissible limit of 85%. The fibers may absorb the moisture, which may lead to binder stripping, causing failure at lower stress. This would be consistent with the hypothesis that this type of fiber improves the performance of the mixture under dry conditions but requires a greater amount of binder to work properly. Therefore, the binder content can be increased considering that there was negligible draindown. 

### 3.4. Toughness

**Figure 6:**
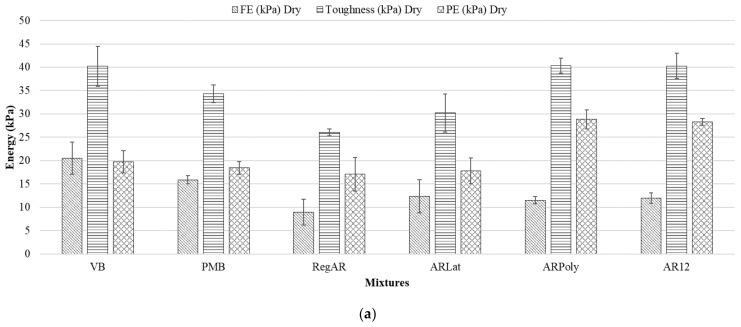

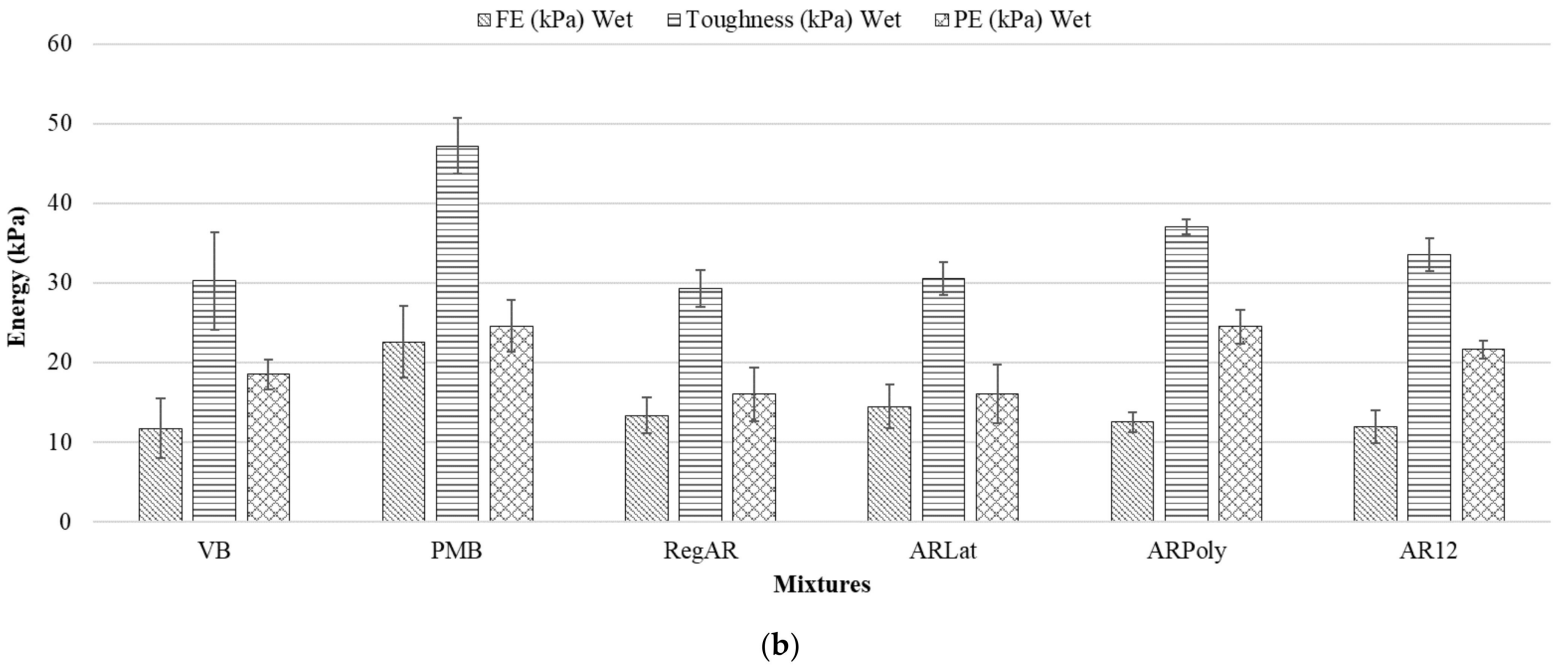
Mean values of fracture energy (FE), post-cracking energy (PE), and Toughness: (**a**) dry conditions (**b**) wet conditions, error bars indicate the standard deviation by mean.

A total of 48 samples were tested for computation of toughness and the ANOVA analysis is shown in Table 7; the mean values of FE, PE, and toughness under dry and wet conditions are given in Figure 6a,b. These graphs were recorded by a data acquisition system under dry and wet conditions.

According to the results, the impact of the fibers differs depending on the conditions. Under dry conditions, RegAR fibers reduce the toughness of the mixtures, with a large reduction in fracture energy FE of 56.6% (*p*-value < 0.05) to VB and 43.6% lower (*p*-value < 0.05) with respect to and PMB, respectively. This indicates their negative influence on the cracking resistance of the mixtures. In the case of PE values, the regular aramid fibers provide 13.4 and 7.3% lower values (*p*-value > 0.05) concerning reference mixtures VB and PMB. ARLat fibers worsen the fracture energy as well (39.51% and 22.1% as compared to VB and PMB respectively). However, the reductions are lower than for regular aramid fibers. Concerning the PE, ARLat performs better than regular fibers, which suggests that the latex coating is suitable to reduce the ductility of the porous asphalt mixtures. Aramid fibers with polyurethane coating result in a reduction of FE and a considerable increase in PE (46.13% and 56.43% as compared to VB and PMB respectively). This suggests that the polyurethane coating improves the ductility of the mixtures. The increment in PE led to a higher value of toughness while using polyurethane coating in dry conditions. AR12 follows a similar trend to ARPoly with significant reductions in fracture energy and significant improvement in post cracking energy. This may be explained by the increment in length from 6 mm in regular fibers to 12 mm in AR12, which may provide more ductility after cracking in mixtures. The toughness of mixtures with aramid fibers of 12 mm length is even higher than PMB in dry conditions. It is worth noting that the toughness of virgin binder was found to be higher than polymer-modified bitumen, which is quite unexpected considering modified bitumen has greater ductility and elasticity that provide higher resistance to fracture.

Concerning wet conditions (see Figure 6b), RegAR has negligible influence on the toughness compared to VB, and as compared to PMB the toughness is lower for both FE (41%) and PE (34.9%). ARLat fibers improved the fracture energy compared to the virgin bitumen (23.9%) but exhibit lower FE than PMB mixtures (22%). However, ARPoly and AR12 fibers improve the PE by 46.2% and 43.6% compared to VB respectively under wet conditions. This confirms their positive effect on the ductility of the mixtures. 

In general, aramid fibers have a negative influence on the toughness of porous asphalt mixtures compared to both virgin binder and polymer-modified bitumen if used with same percentage of bitumen. The reductions in energy parameters are noteworthy which is in agreement with the previous study that utilized aramid plus polyolefin fibers [19]. It was concluded that the effects as compared to the control mixtures are not significant. However, the fibers were not able to reach the performance of PA mixtures with PMB, as these fibers have shown adverse effects on the moisture resistance of mixtures and the toughness under wet conditions. Regular aramid fibers of 6 mm displayed the worst performance in toughness as compared to PMB under wet conditions. However, aramid fibers ARPoly have greater toughness compared to RegAR. ARPoly displays high toughness due to its polyurethane coating and AR12 fiber improves the ductility due to the greater length of the fiber.

## 4. Conclusions

In this study, the effect of different aramid fibers on the mechanical resistance of the porous asphalt mixtures was evaluated by laboratory testing (permeability test, Cantabro test, indirect tensile strength test, and moisture susceptibility test). The porous asphalt mixtures were designed with a 4.5% binder content of virgin bitumen and polymer-modified bitumen, and fiber content of 0.05% was fixed to analyze the influence of the different fibers. Four types of aramid fibers including regular aramid fibers of 6 mm and 12 mm length; aramid fiber with latex and polyurethane coatings were added in open-graded mixtures. The following conclusions can be drawn from the laboratory testing and statistical analysis of the results:

- With this percentage of binder, the addition of fibers had a negligible influence on the permeability of porous asphalt mixtures. The air void contents of all the mixtures with fibers were quite similar and exceeded 20%.
- Aramid fibers enhanced the abrasion resistance of the mixtures. ARLat fibers displayed the best performance with similar results to PMB. While ARPoly reduced the abrasion resistance of the mixtures under wet conditions.
- All aramid fibers were shown to have a positive effect on the dry ITS values of porous asphalt mixtures, although the observed increment was low. Particularly, ARPoly and RegAR fibers improve the ITS under dry conditions by up to 8% and 10%, respectively.
- Under wet conditions, the fibers considerably reduce the ITS of porous asphalt mixtures. This may be because of the high susceptibility of aramid fibers to the presence of moisture or to requiring higher bitumen content. Additionally, it was found that the polymer coating on fibers minimized the damage caused due to penetration of moisture; as coated fibers (ARPoly and ARLat) showed better results in comparison to uncoated aramid fibers.
- Concerning the post cracking energy, significant improvement was observed by incorporation of polyurethane coating (ARPoly) and use of higher length fibers (AR12) fibers under dry conditions. The possible explanation may be polyurethane coating increases the durability while a higher length of fibers of AR12 facilitates more deformation at the same stress. Meanwhile, shorter length (RegAR) and latex coated fibers ARLat had adverse effects on post cracking energy.
- Aramid fibers had a negative impact on the fracture energy of porous asphalt mixtures in dry conditions, but their impact is negligible under wet conditions. The fibers did not achieve the behavior of PMB.
- Overall, aramid fibers were found to be susceptible to moisture, which is reflected by reduction in ITS and toughness of the mixtures under wet conditions. Higher percentages of binder would be required to facilitate the strengthening and anti-drainage capacity of the aramid fibers.

The conclusions were drawn based on permeability, particle loss, indirect tensile testing, and energy parameters. However, more research is required to evaluate the impact of aramid fibers on the fatigue behavior and low temperature cracking resistance of porous asphalt mixtures. Moreover, it is necessary to evaluate these results with a higher binder content, which may lead to a reduction in moisture susceptibility.

## Figures and Tables

**Table 1:** Properties of bitumen used in the study.

Properties	Standard	Virgin Binder 50/70	PMB 45/80-65
Relative Density	EN 15326	1.035	1.028
Penetration (0.1 mm)	EN 1426	57	55
Softening point (°C)	EN 1427	51.6	74.1
Frass point (°C)	EN 12593	−13	-
Elastic Recovery at 25 °C (%)	EN 13398	-	92

**Table 2:** Properties of fibers and aggregates used in the study.

		**Fibers**		
**Properties**	**RegAR**	**ARLat**	**ARPoly**	**AR12**
Coating	none	Resorcinal formaldehyde latex (RFL)	Polyurethane	none
Density (g/cc)	1.39	1.39	1.44	1.44
tensile strength (GPa)	3.2–3.5	3.2–3.5	2.7–3.6	2.7–3.6
Length (mm)	6	6	6	12
Moisture (%)	1.9	1.9	2.7	2.7
**Mineral Aggregates**				
**Properties**	**Standard**	**Coarse Aggregates**	**Fine Aggregates**	**Limits**
Specific Weight (g/cm3)	EN 1097-6	2.787	2.705	-
Los Angeles (%)	EN 1097-2	15	-	≤15%
Flakiness Index (%)	EN 933-3	12	-	≤20%
Sand equivalent (%)	EN 933-8	-	78	>55

**Table 3:** Porous asphalt mixtures used in the study.

Short Name of Mixtures	Bitumen	Fiber Content (by wt. of Mix)	Bitumen Content (by wt. of Mix)
VB	Virgin binder 50/70	-	4.5
PMB	45/80-65	-	
RegAR	Virgin binder 50/70	0.05	
ARLat	Virgin binder 50/70	0.05	
ARPoly	Virgin binder 50/70	0.05	
AR12	Virgin binder 50/70	0.05	

**Table 4:** Minimum permeability.

Authors	Minimum Permeability (mm/day)	Standards
Cetin 2013 [47]; Andres-Valeri 2018 [48]	1.15	ASTM D7064-04, ASTM D3637
Sangiorgi 2016 [49]	0.5	Italian technical specifications
ASCE 2013 [50]	1.2	-
Mallick et al. [51]	1.16	-

**Table 5:** Statistical analysis for the abrasion resistance (parametric test).

Mixture	*p*-Value	Group	Percent Change w.r.t VB (%)	Percent Change w.r.t PMB (%)
			Dry conditions	
VB	<0.05	A	-	-
PMB		A, B	-	-
RegAR		A	↑ 7.2	↓ 31.65
ARLat		B	↑ 46.9	↑ 24.72
ARPoly		A	↑ 5.3	↓ 34.29
AR12		A	↑ 8.7	↓ 29.57
			Wet conditions	
VB	<0.05	A, B	-	-
PMB		B	-	-
RegAR		A, B	↑ 8.99	↓ 42.21
ARLat		B	↑ 32.60	↓ 5.32
ARPoly		A	↓ 29.59	↓ 102.50
AR12		A, B	↑ 11.24	↓ 38.70

Note: The upward symbol indicates improvement and downward shows a reduction in abrasion resistance.

**Table 6:** Results of ANOVA analysis for indirect tensile strength (parametric test).

Mixture	*p*-Value	Group	Percent Change w.r.t VB (%)	Percent Change w.r.t PMB (%)
			Dry conditions	
VB	<0.05	A	-	-
PMB		A, B	-	-
RegAR		B, C	↑ 8.3	↓ 0.1
ARLat		C	↑ 2.9	↓ 5.1
ARPoly		A, B,	↑ 10	↑ 1.4
AR12		A, B, C	↑ 5.8	↓ 2.4
			Wet conditions	
VB	<0.05	A	-	-
PMB		A	-	-
RegAR		C	↓ 27.3	↓ 32.9
ARLat		B, C	↓ 20.6	↓ 26.7
ARPoly		B	↓ 15.5	↓ 22.1
AR12		C	↓ 27.7	↓ 33.3

Note: The upward arrow indicates improvement and the downward arrow shows a reduction in indirect tensile strength.

**Table 7:** Results of ANOVA analysis for toughness (parametric test).

Mixture	Group	Percent Change w.r.t VB (%)	Percent Change w.r.t PMB (%)
		Dry conditions	
VB	A, B	-	-
PMB	B, C	-	-
RegAR	A, B	↓ 35.23	↓ 24.14
ARLat	C	↓ 24.86	↓ 12.00
ARPoly	A	↑ 0.32	↑ 17.50
AR12	A	↑ 0.22	↑ 17.38
		Wet conditions	
VB	A, B	-	-
PMB	A	-	-
RegAR	D	↓ 3.14	↓ 37.89
ARLat	C	↑ 1.02	↓ 35.23
ARPoly	B, C	↑ 22.39	↓ 21.52
AR12	C	↑ 10.91	↓ 28.89

## Data Availability

Not applicable.
